# Targeted Athletic Training Improves the Neuromuscular Performance in Terms of Body Posture From Adolescence to Adulthood – Long-Term Study Over 6 Years

**DOI:** 10.3389/fphys.2018.01620

**Published:** 2018-11-27

**Authors:** Oliver Ludwig, Jens Kelm, Annette Hammes, Eduard Schmitt, Michael Fröhlich

**Affiliations:** ^1^Technische Universität Kaiserslautern, Fachgebiet Sportwissenschaft, Kaiserslautern, Germany; ^2^Chirurgisch-Orthopädisches Zentrum, Illingen, Germany; ^3^MEDICOVER GmbH, Saarbrücken, Germany; ^4^Universitätsklinikum des Saarlandes, Klinik für Orthopädie und Orthopädische Chirurgie, Homburg, Germany

**Keywords:** posture analysis, posture training, adolescence, maturation, posture development, body perception

## Abstract

Poor posture in childhood and adolescence is held responsible for the occurrence of associated disorders in adult age. This study aimed to verify whether body posture in adolescence can be enhanced through the improvement of neuromuscular performance, attained by means of targeted strength, stretch, and body perception training, and whether any such improvement might also transition into adulthood. From a total of 84 volunteers, the posture development of 67 adolescents was checked annually between the age of 14 and 20 based on index values in three posture situations. 28 adolescents exercised twice a week for about 2 h up to the age of 18, 24 adolescents exercised continually up to the age of 20. Both groups practiced other additional sports for about 1.8 h/week. Fifteen persons served as a non-exercising control group, practicing optional sports of about 1.8 h/week until the age of 18, after that for 0.9 h/week. Group allocation was not random, but depended on the participants’ choice. A linear mixed model was used to analyze the development of posture indexes among the groups and over time and the possible influence of anthropometric parameters (weight, size), of optional athletic activity and of sedentary behavior. The *post hoc* pairwise comparison was performed applying the Scheffé test. The significance level was set at 0.05. The group that exercised continually (TR20) exhibited a significant posture parameter improvement in all posture situations from the 2nd year of exercising on. The group that terminated their training when reaching adulthood (TR18) retained some improvements, such as conscious straightening of the body posture. In other posture situations (habitual, closed eyes), their posture results declined again from age 18. The effect sizes determined were between η^2^ = 0.12 and η^2^ = 0.19 and represent moderate to strong effects. The control group did not exhibit any differences. Anthropometric parameters, additional athletic activities and sedentary behavior did not influence the posture parameters significantly. An additional athletic training of 2 h per week including elements for improved body perception seems to have the potential to improve body posture in symptom free male adolescents and young adults.

## Introduction

Upright posture is the result of a complex interaction of skeleton, musculature, and central nervous system (CNS) (Assaiante et al., 2005). Neuromuscular performance determines the quality of posture and movement control, which is key for mastering daily routine tasks and athletic activity (Punakallio, 2003). It changes in the course of time, which is based on increasing muscular weakness and control deficits in the CNS. Already in childhood and adolescence, deficits in posture control have an effect, usually in the form of weak posture. Typical weaknesses are, for example, lumbar hyperlordosis, hunchback, protracted shoulders, and protruded head. Depending on the definition of posture weaknesses, literature states their prevalence at 22–65% for 10- to 18-year-old children and adolescents (Kratenova et al., 2007; Gh Maghsoud et al., 2012; Wirth et al., 2013; Lee, 2016).

The connection between posture weaknesses and the occurrence of complaints in adolescence or in the course of adulthood has not been clarified to date. However, more and more studies suggest a link to back and neck pain during adolescence and in the course of later development (Dolphens et al., 2015, 2016; Noll et al., 2016). It is assumed that posture weaknesses may lead to a biomechanically unfavorable strain of tendons and joints, and that corresponding adaptations result in asymmetrical muscle activity, which, in turn, leads to muscular problems (Bruno et al., 2012).

If posture weakness in adolescents could lead to problems in adulthood, early intervention is required. Since posture weakness is often accompanied by weak muscle function (Buchtelová et al., 2013), the usual intervention - depending on the severity of problems – is physical therapy, rehabilitation sport, or a recommendation to increase athletic activity in general (Kim et al., 2015).

This recommendation becomes ever more important because the lifestyle of children in industrialized nations has changed to be more sedentary and less active (Claus et al., 2009; Drzal-Grabiec and Snela, 2012; Shan et al., 2014). Therefore, targeted athletic activity seems to be a suitable means to preventing posture problems. However, we need to critically question (i) whether targeted athletic activity in adolescence operates as a preventive or corrective factor in terms of posture deficits, and (ii) whether athletic activity in adolescence has positive effects reaching all the way into adulthood. According to current knowledge, targeted strength training can be performed already in childhood and adolescence [overview in (Matos and Winsley, 2007)].

The extent to which targeted posture training in adolescence carries positive effects into adulthood, i.e., the question whether it basically lays the foundation for a stable body posture, has not been clarified to date. Therefore, this study meant to verify whether a targeted training program in adolescence can carry positive effects into adulthood. The following hypotheses were to be analyzed:

(i) Targeted posture training improves selected posture parameters in adolescence.
(ii) Posture training performed on a regular basis in adolescence continues to have positive effects on body posture in adulthood.

## Materials and Methods

### Sample

The study was carried out within the framework of an interdisciplinary research project (Kid-Check). As the primary goal was to examine intra-individual changes during a perennial training program, the minimal sample size was calculated with G^∗^Power 2.1 (University Kiel, Germany) with alpha = 0.05, power = 0.95, effect size = 0.5, based on matched pairs *T*-test. We calculated a total sample size of 45, but increased this due to an expected high number of dropouts.

**FIGURE 1:**
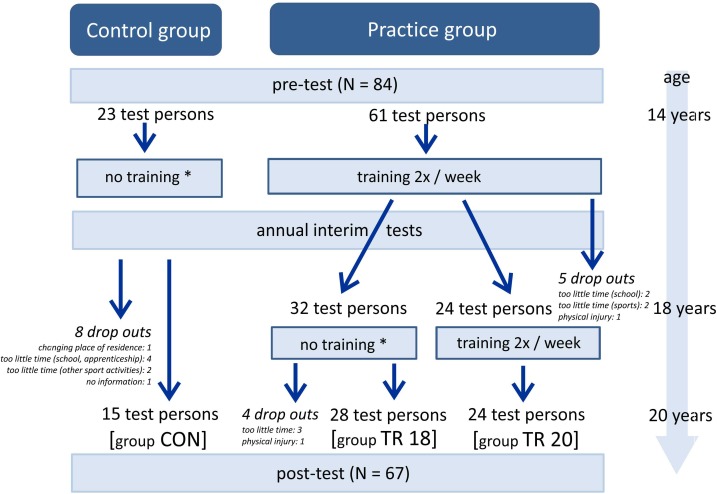
Study flow chart. ^∗^Reasons see text.

**FIGURE 2:**
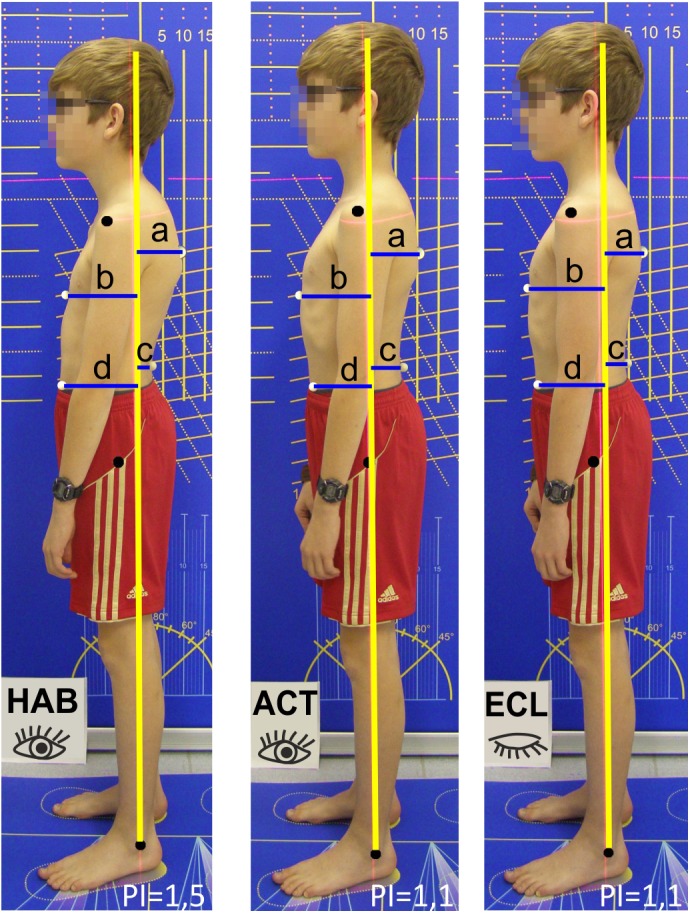
Schematic posture analysis in the three posture situations habitual (HAB), active with open eyes (ACT), active with closed eyes (ECL). The posture indices HI are calculated as (a+d)/(b+c). Photomontage, courtesy of Elsevier publishing house.

Between 2001 and 2018, a total of 84 adolescent male test persons with poor posture participated in the study (see flow chart, Figure 1). The adolescents (please refer to Table 1 for anthropometric data) entered the study at age 14, and 67 of them were examined annually until the age of 20. The key criterion to be included in the study was a significant posture problem, defined by means of a posture index pertaining to habitual posture > 1.35 [Figure 2 (Fröhner, 1998; Ludwig et al., 2016c)]. Exclusion criteria were acute complaints pertaining to the postural and musculoskeletal system, pathological changes in the spine, a BMI > 24 or an intensive (>3 h per week) additional athletic activity.

**Table 1:** Anthropometric data and leisure behavior of the test groups at the start of the study.

	Mass (kg)	Height (cm)	Athletic activity (h/week)	Sedentary behav. (h/week)
TR20	60.3 ± 4.96	171.0 ± 5.49	1.87 ± 1.42	38.54 ± 5.32
TR18	60.9 ± 5.00	171.8 ± 4.28	1.86 ± 1.51	37.94 ± 4.50
CON	60.5 ± 5.76	171.6 ± 6.03	1.90 ± 0.87	39.37 ± 4.87


Twenty-three of the 84 test persons served as a control group. They were not randomly chosen. Instead, we added all persons to the control group who could not guarantee a regular participation in a two-times-per-week training. The main reasons were: too little time (caused by school, *N* = 4, caused by leisure activities, *N* = 5) and logistics problems regarding their transportation to the training location (*N* = 12). Two participants were not willing to perform a regularly strength training.

The later division of the training group at age 18 was not random, either. Initially, we planned to terminate the study when the participants came of age, but later decided to continue for two further years, if a sufficient number of participants would continue. The 28 test persons who stopped training did so for different reasons: too little time caused by school or studies (*N* = 16), changing their place of residence due to university studies (*N* = 6), having reached their personal fitness goals (*N* = 4), and physical injury (*N* = 2). According to them, none of them stopped because they had lost their interest in strength training or due to lack of motivation.

During the 6 years, a total of 17 persons dropped out at different times. Their reasons for leaving the study are indicated in Figure 1. From that point onward, they were no longer available for any subsequent tests.Nevertheless, in order to reduce bias, we still included their data in the analysis up to the date of them having left the study.

The study’s concept was based on the Helsinki declaration and conducted accordingly (World Medical Association, 2013). The university’s ethics commission had approved the study (ref. no. 15-6). The test persons and their parents were informed on the order of the study and the content of the training and gave their written consent.

### Posture Analysis

Since body posture is a complex phenomenon it was operationalized through a sum parameter [posture index (Fröhner, 1998; Ludwig et al., 2016b)] in three different posture situations in order to provide an overview of the general posture control (Woollacott and Shumway-Cook, 1990; Assaiante et al., 2005; Maurer et al., 2006; Ludwig et al., 2016d).

The following posture situations were registered for posture identification and comparison at an examination day once a year: the habitual, relaxed position (HAB), the active, upright position (ACT), and the active upright position with closed eyes (ECL) (Ludwig et al., 2016d). While active posture can be reproduced validly (Ludwig et al., 2016b), habitual posture changes during the day. To improve internal validity, all measurements were taken at the same time in the morning without any previous intensive physical activity, and followed a standardized test protocol (Ludwig et al., 2016d).

To determine the posture parameters, posture photographs in the sagittal plane were taken of the adolescents wearing swimwear or underwear. High-contrast marker balls were attached to anatomic landmarks as the caudal tip of the sternum, the point of the strongest lumbar lordosis, the point of the strongest thoracic kyphosis, as well as the spina iliaca anterior superior. A camera was mounted on a tripod at hip height (Olympus SP510UZ and Nikon Coolpix S33) and posture photographs (resolution 2304 pixels × 3072 pixels) of all three posture situations were taken in front of a calibration wall.

The adolescents first stood relaxed, feet at shoulder width, arms hanging down loosely, view straight ahead. A sideways photograph was taken (HAB). The adolescents were then instructed to actively change into an upright position without holding their breath. A second posture photograph was taken (ACT). The adolescents were then instructed to close their eyes while maintaining their active posture. After 60 s, the third photo was taken (ECL). The instructions were standardized and no optical or acoustic disruptive impulses occurred. All posture analyses were performed by an experienced researcher who was blinded to the group membership of the participants.

The horizontal distances between marker points and the perpendicular through the malleolus lateralis were calculated using the Corpus concepts^®^ software (Fa. AFG, Idar-Oberstein, Germany), and the posture index was calculated based on those results. The posture index is a complex parameter that summarily evaluates the posture quality of the trunk (for details please see Figure 2). Values between 1.0 and 1.3 stand for a stable posture (Fröhner, 1998; Ludwig et al., 2016c). The test quality of this parameter has been confirmed in other studies (Ludwig et al., 2016b). The advantage of this parameter for the assessment of body posture is that it combines numerous individual posture parameters into one numerical value. Current studies show that the “global” posture parameters are associated with complaints, while “local” parameters, such as the pelvic angle, do not provide clear information (Dolphens et al., 2015).

### Posture Training

From the start of the study, the 61 adolescents exercised for 60 min twice a week in a gym under qualified supervision. After a 6-min warm-up phase on a treadmill, they performed the strength-endurance exercises in the form of a set-of-3 training at the device (15 repetitions, 1 min break, see Table 2). During the 1st months, the participants familiarized themselves with the training devices. The weight load was kept low and the participants’ awareness was led to focus on proper movement. The weights were chosen and adapted during the study so that the participants were able to carry out the 3 sets while adhering to the correct movement technique, but feeling definitely exhausted subjectively afterwards (Borg scale 7 of 10). The stretch exercises (active antagonist contract stretching) were executed three times each for 30 s for each side of the body (Youdas et al., 2010). As the main reason for poor posture is found in poor motor skills and weakness of the supporting musculature, e.g., due to sedentary day-to-day school life, neuromuscular performance can be improved via multiple approaches:

**Table 2:** Exercises of the multi-dimensional posture training program.

	Training goals		Target muscle/*movement objective*	Equipment/Position	Movement
Strength	*Strengthening*	1	M. glutaeus max.	Glutaeus machine	Move a straight leg backward against resistance
		2	M. biceps fem.	Knee flexion, sitting	While seated with the knees at a right angle move the calves backward against resistance
		3	M. rectus abdom.	*Abdominal machine*, sitting	While seated bend the upper body forward against resistance
Stretching/Mobility	*Stretching/improving range of motion (ROM)*	4	M. iliopsoas, M. quadriceps fem.	Antagonist contract stretching in a lying position	Move the straight leg actively, then passively backward in the hip joint
		5	M. rectus femoris	Antagonist contract stretching in a lunge	Move the rear knee actively backward toward the lower back, then passively using a hand for support
Body perception	*Body perception*	6	*Control of pelvic position/Reducing lumbar lordosis*	Supine position	Actively neutralize lumbar lordosis under muscular tension
		7	*Control of pelvic position*	Supine position	Thigh vertical, knee bent at 90°, slightly (1 cm) lift pelvis from the floor
		8	*Control of pelvic position/Global posture*	Standing position	Tilt pelvis backward and forward, upper body and thighs remain motionless
		9	*Posture correction*	Standing position with mirror control	Targeted alignment of the body with the perpendicular

- Strengthening the weak core muscles (strength endurance)
- Improving the range of motion (ROM) of the movement-limiting muscles (mobility/flexibility)
- Improving the sensory-neuromuscular coordination.

The focus of the posture training therefore lied in three dimensions (Table 2):

(1) Strengthening the muscle groups that straighten up the pelvis (in particular, m. rectus abdominis, m. obliquus, hamstrings, and m. glutaeus maximus) because they are able to effect an active retroversion of the pelvis (Buchtelová et al., 2013; Jeong et al., 2015).
(2) Stretching the muscles involved in the forward tilt of the pelvis (m. iliopsoas, m. rectus femoris) in order to increase the ROM during pelvic retroversion (López-Miñarro et al., 2012; Konrad and Tilp, 2014). The antagonist-contract stretching (AC stretching) employed is known as an established method to increase the ROM and has the added advantage of easy and unsupported implementation (Konrad et al., 2017).
(3) Exercises for body perception (pelvis lift lying down, lordosis adjustment, pelvis retroversion, perception of pelvic movement) (Bruhn et al., 2004). These exercises especially trained the self-perception of body posture and active use of the muscle groups that straighten the pelvis.

Details of the athletic posture training were presented in an earlier study (Ludwig et al., 2016a). Every training session was protocolled in a person-specific, paper-based training protocol, in which the number of repetitions and the individual loads were noted. All training sessions were instructed by the same trainer (first author O.L.), who supervised correct movements and motivated the participants if necessary. Overall, the participants’ training attendance was very good: as long as they participated in the study their average posture training workload was 1.81 h/week.

The posture measurements were repeated annually. After reaching adulthood, 32 adolescents left the posture training at their own request. 28 of these, however, still participated in the annual control examinations. 24 adolescents continued to exercise weekly until they reached age 20 (see flowchart in Figure 1). During the annual examinations, the weekly sedentary and standing behavior and athletic activity were recorded using a survey in form of a questionnaire (Supplementary Data Sheet 1). This served to identify and evaluate potential disturbance factors arising from the now non-school daily routines, which differed greatly among the test persons.

### Statistics

Potential differences between the groups TR18 (training until the age of 18, then stop), TR20 (uninterrupted training until the age of 20), and CON (control group) before training start were examined by means of univariate variance analysis (ANOVA) for anthropometric parameters, athletic activity, sedentary parameters, and the posture indexes of the HAB, ACT, and ECL posture situations.

In order to identify potential confounding effects caused by anthropometric parameters or leisure behavior, we used a linear mixed model approach (mixed design ANOVA) with the posture indexes as dependent variables, and body weight, body height, hours of athletic activity per week, hours of sedentary activity, time, and group as model variables. We included all available data in this model. The homogeneity of the variances was verified using the Levene test, the heteroscedasticity was tested by the modified Breusch–Pagan-test.

*Post hoc* pairwise comparisons were performed according to Scheffé. The effect size was estimated based on Cohen’s effect size measures based on partial eta square (η^2^) and Cohen’s *d* (Fröhlich and Pieter, 2009). A strong effect exists with η^2^ ≥ 0.14. The significance level was set at 0.05.

## Results

**FIGURE 3:**
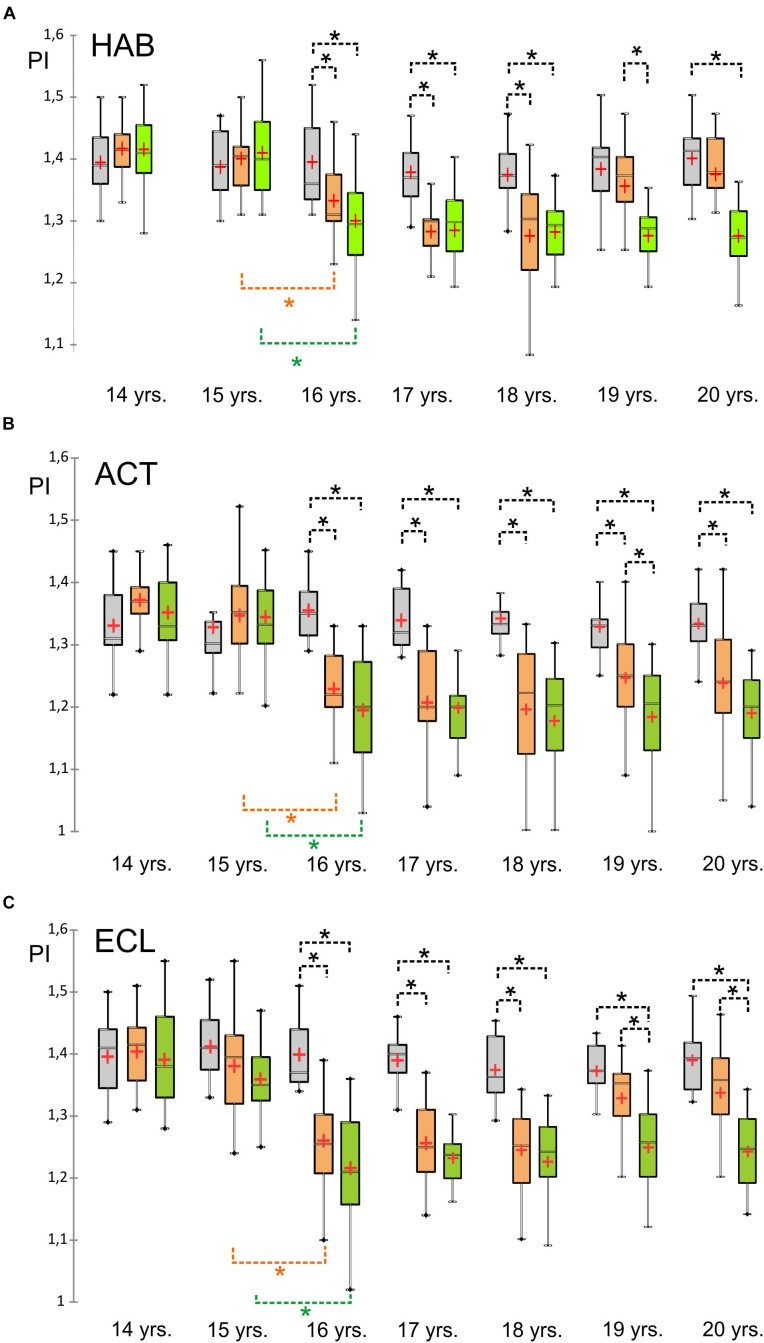
Box plots of the posture index value development for the three groups over time. **(A)** (Above): habitual posture, **(B)** (middle): active posture with open eyes, **(C)** (below): active posture with closed eyes. *Y*-axes: posture index PI. Green: TR20, orange: TR18, gray: CON; ^∗^ indicate significant differences.

When the study started (age 14), there were no significant differences in the anthropometric data among the three groups (weight: *F* = 0.27, df = 2, *p* = 0.77, height: *F* = 0.22, df = 2, *p* = 0.80). The parameters describing leisure behavior, like sedentary behavior (*F* = 0.74, df = 2, *p* = 0.48) and athletic activities (*F* = 0.01, df = 2, *p* = 0.99) did not differ significantly, as well as the posture indices for habitual posture (HAB, *F* = 0.84, df = 2, *p* = 0.44), active posture (ACT, *F* = 2.72, df = 2, *p* = 0.07), and active posture with closed eyes (ECL, *F* = 0.29, df = 2, *p* = 0.75). Data are presented in Tables 1, 3 and the development over time is indicated in Figure 3.

**Table 3:** Posture parameter development in the three posture positions and test groups over time.

			TR20				TR18				CON		
		*n*	Mean	*SD*	C.I. (95%)	*n*	Mean	*SD*	C.I. (95%)	*n*	Mean	*SD*	C.I. (95%)
**Habitual**	14 years	26	1.42	0.06	1.39–1.44	31	1.42	0.05	1.40–1.44	23	1.41	0.06	1.36–1.43
	15 years	26	1.40	0.07	1.38–1.44	31	1.40	0.06	1.38–1.42	23	1.40	0.06	1.36–1.42
	16 years	26	1.30	0.08	1.27–1.34	31	1.33	0.07	1.31–1.36	22	1.40	0.07	1.36–1.43
	17 years	25	1.28	0.05	1.26–1.30	30	1.28	0.05	1.26–1.30	19	1.39	0.06	1.35–1.41
	18 years	24	1.28	0.05	1.26–1.30	28	1.27	0.09	1.24–1.31	16	1.37	0.05	1.34–1.40
	19 years	24	1.27	0.06	1.25–1.30	28	1.36	0.08	1.33–1.39	16	1.39	0.07	1.35–1.42
	20 years	24	1.27	0.05	1.25–1.30	28	1.37	0.08	1.34–1.40	15	1.40	0.06	1.37–1.43
**Active, eyes open**	14 years	26	1.35	0.06	1.33–1.38	31	1.37	0.05	1.35–1.39	23	1.32	0.05	1.30–1.36
	15 years	26	1.34	0.07	1.31–1.37	31	1.34	0.08	1.31–1.38	23	1.32	0.07	1.28–1.37
	16 years	26	1.20	0.08	1.16–1.23	31	1.23	0.06	1.20–1.25	22	1.34	0.05	1.33–1.38
	17 years	25	1.20	0.06	1.17–1.22	30	1.21	0.08	1.18–1.24	19	1.34	0.05	1.31–1.37
	18 years	24	1.18	0.09	1.14–1.21	28	1.19	0.10	1.15–1.23	16	1.34	0.05	1.31–1.37
	19 years	24	1.18	0.09	1.15–1.22	28	1.25	0.07	1.22–1.27	16	1.33	0.05	1.30–1.36
	20 years	24	1.19	0.07	1.16–1.22	28	1.24	0.11	1.20–1.28	15	1.33	0.05	1.30–1.36
**Active, eyes closed**	14 years	26	1.39	0.07	1.36–1.42	31	1.40	0.05	1.38–1.42	23	1.40	0.07	1.36–1.43
	15 years	26	1.36	0.06	1.33–1.38	31	1.38	0.07	1.35–1.41	23	1.41	0.06	1.38–1.44
	16 years	26	1.22	0.09	1.18–1.25	31	1.26	0.07	1.23–1.29	22	1.40	0.06	1.37–1.43
	17 years	25	1.23	0.06	1.20–1.26	30	1.26	0.06	1.23–1.28	19	1.39	0.04	1.37–1.41
	18 years	24	1.22	0.07	1.19–1.25	28	1.24	0.07	1.22–1.27	16	1.37	0.06	1.34–1.40
	19 years	24	1.25	0.08	1.21–1.28	28	1.33	0.07	1.30–1.35	16	1.37	0.05	1.34–1.40
	20 years	24	1.24	0.06	1.22–1.27	28	1.33	0.07	1.31–1.36	15	1.39	0.05	1.36–1.41


*SD, standard deviation; C.I., 95% confidence interval.*

After 2 years of training (age 16), we found significant improvements in the exercising groups TR18 and TR20 for all posture positions (posture index < 1.35, *F* = 7.62, df = 2, *p* < 0.001), both within the group and in comparison with the control group. From age 18 on, the posture parameters changed in different ways, which we will describe in the following paragraphs.

### Habitual Posture

The linear mixed model showed a significant inter-individual effect for the *Group* factor for the habitual posture in the course of the study (*F* = 52.107, df = 2, *p* < 0.0001, η^2^ = 0.18) and significant intra-subject effects for the *Time* factor (*F* = 13.345, df = 6, *p* < 0.0001, η^2^ = 0.14) and the interaction *Group^∗^Time* (*F* = 7.059, df = 12, *p* < 0.0001, η^2^ = 0.15).

The other model variables did not show any significant effect (body weight: *F* = 1.485, df = 1, *p* = 0.224; height: *F* = 0.008, df = 1, *p* = 0.927; hours of athletic activity: *F* = 3.532, df = 1, *p* = 0.061; hours of sedentary activity: *F* = 1.724, df = 1, *p* = 0.190).

After the study was terminated, group comparisons of posture indexes between control group and TR 18 (*p* = 0.06) and between TR18 and TR20 (*p* = 0.10) showed no significant pair differences, while the control group and the TR20 group differed significantly (*p* < 0.001). During the joint training phase up to age 18, no significant differences between the groups TR18 and TR20 were identified. In the course of the study, the TR18 group came closer to the control group after having suspended their exercises, while the TR20 group continued to improve their habitual posture (*p* < 0.001, *d* = 1.2) (Figure 3A).

### Active Posture

For active posture, significant inter-individual effects were identified for the *Group* factor (*F* = 69.701, df = 2, *p* < 0.0001, η^2^ = 0.225), in addition to significant intra-individual effects for the *Time* factor (*F* = 10.918, df = 6, *p* < 0.0001, η^2^ = 0.120) and the interaction *Group^∗^Time* (*F* = 9.171, df = 12, *p* < 0.0001, η^2^ = 0.187). *Post hoc* group comparisons after the end of the study showed significant differences between the two training groups and the control group (*p* < 0.001 each).

The linear mixed model did not show any significant effect for the other model variables (body weight: *F* = 2.189, df = 1, *p* = 0.140, height: *F* = 0.259, df = 1, *p* = 0.611; hours of athletic activity: *F* = 0.017, df = 1, *p* = 0.897; hours of sedentary activity: *F* = 0.320, df = 1, *p* = 0.572). During the intervention phase, the two training groups did not differ significantly (15 years: *p* = 0.99, 16 years: *p* = 0.19, 17 years: *p* = 0.88, 18 years: *p* = 0.73). After the training was suspended by TR18 at age 18, the groups TR18 and TR20 differed in the 1st year (19 years: *p* = 0.01), while no difference was found in the 2nd year (20 years: *p* = 0.10) (Figure 3B).

### Active Posture With Closed Eyes

For active posture with closed eyes, significant inter-individual effects were found for the *Group* factor (*F* = 111.517, df = 2, *p* < 0.0001, η^2^ = 0.318), in addition to significant intra-individual effects for the *Time* factor (*F* = 22.427, df = 6, *p* < 0.0001, η^2^ = 0.219) and the interaction *Group^∗^Time* (*F* = 8.171, df = 12, *p* < 0.0001, η^2^ = 0.170). The two intervention groups differed from the control group (*p* < 0.001 each) in the active posture with closed eyes. From age 19, a significant difference was identified between TR18 and TR20 (19 years: *p* < 0.001, 20 years: *p* < 0.0001), while no difference was found between CON and TR18 (Figure 3C).

All other model variables did not show any significant effect (body weight: *F* = 1.032, df = 1, *p* = 0.310, height: *F* = 0.307, df = 1, *p* = 0.580; hours of athletic activity: *F* = 0.293, df = 1, *p* = 0.589; hours of sedentary activity: *F* = 0.017, df = 1, *p* = 0.895).

### Sedentary Behavior and Athletic Activity

**FIGURE 4:**
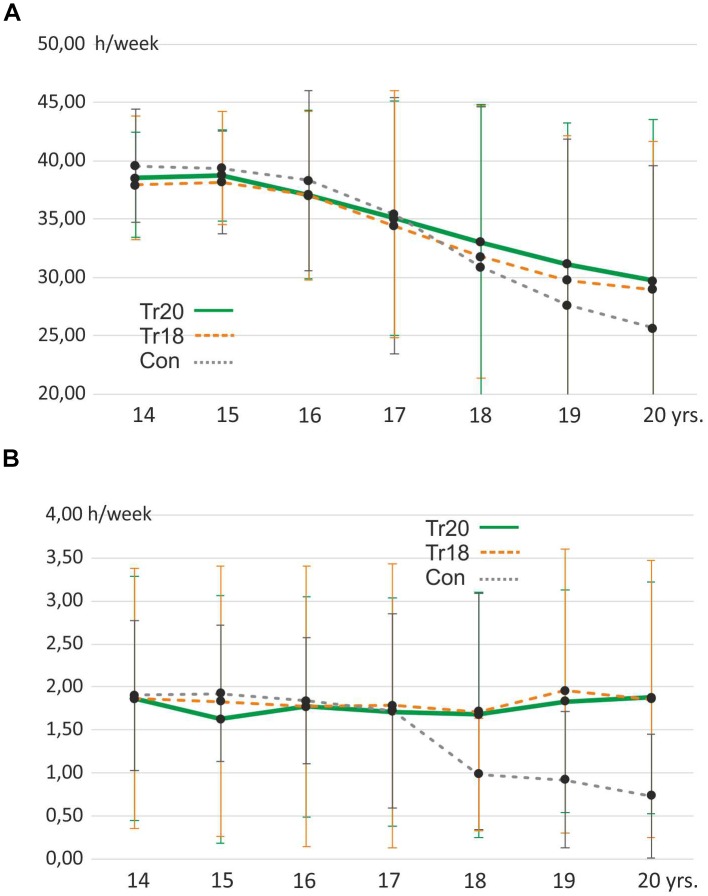
Development of sedentary behavior **(A)** and supplemental athletic activities **(B)** in hours per week of the three groups over time (mean values, bars represent standard deviations).

In the course of the study, the weekly time spent in a sitting position was comparable between the three groups (Figure 4A). At the end of the study, all three groups did not differ significantly in terms of sedentary behavior (*F* = 0.46, df = 2, *p* = 0.63) or standing behavior (*F* = 0.11, df = 2, *p* = 0.90) during school, work, or studies. Athletic activity resulted in significant differences between TR20 (3.59 ± 1.40 h/week) on the one hand, and TR18 (1.39 ± 1.19 h/week) and CON (0.53 ± 0.66 h/week) on the other at the end of the study. When athletic activity was adjusted so that only supplementary activity was analyzed – that is, any activity in addition to the weekly posture training – there were no significant differences between the groups, although it was obvious that the participants of the control group spent less time with athletic activities from age 18 on (Figure 4B). Even though the differences did not reach significance level, their mean time spent with athletic activities was considerably lower.

## Discussion

The objective of this study was to analyze changes in the neuromuscular performance of posture regulation from adolescence to adulthood, and to determine the influence and potential effects of target-oriented athletic posture training.

Body posture is the result of a complex interaction of active structures (receptors and muscles) and passive elements (bones, tendons, fasciae, and ligaments) (Sousa et al., 2012). The quality of body posture control depends on the neuromuscular performance (Kilby et al., 2015).

### Anthropometric Parameters and Leisure Behavior

Analyzing the changes of posture parameters during the long time span of 6 years may be susceptive to many confounders. Anthropometric parameters, such as body weight and height, as well as leisure behavior, such as athletic activities and sedentary behavior, change during the years of physical development and might constitute confounding variables. The latter are not easy to capture, much less to control. We did not find any significant influence of these factors, but we are aware that a survey once a year might not be optimal. Furthermore, more influencing variables like socioeconomic factors, developmental stage, environmental factors, and psychological variables may exist that we did not capture (Brodersen et al., 2005). Therefore, our results need to be interpreted with care.

Anthropometric variables seem not to have influenced posture development in a group-specific manner. The development of body weight and height was similar in all three groups and were within the normal national ranges (Neuhauser et al., 2013). Leisure behavior may also have had an important impact on the adolescents’ physiological status as it is known that teenagers who prefer a more sedentary leisure behavior tend to have weaker muscles and a higher body mass index (Hanson and Chen, 2007). Once a year, we asked for leisure behavior in the form of a questionnaire. Up to the age of majority, this questionnaire was answered by children and parents together to avoid any type of bias that could have been produced by children who wanted to present themselves in a more positive light than was realistic. However, we had to rely on the truthfulness of the given statements. Furthermore, we must be aware that all statements had to be averaged over the period of 1 year. Changes in sedentary behavior during the year may, for example, have been caused by changing to another type of school or attending different classes with a higher or lower number of weekly lessons. For organizational reasons interviews in a shorter period were not feasible. Therefore, these statements need to be considered with caution.

### Effects of Posture Training in Adolescence

Our first hypothesis that a supplemental targeted posture training program may improve selected posture parameters in adolescence seems to be confirmed. The exercising groups TR18 and TR20 showed significantly improved posture parameters after 2 years. The question arises whether the improvement was primarily due to the training program or confounded by other variables. We were able to exclude the influence of body mass and height by applying our mixed-model approach, as explained above. Other supplementary athletic activities did not significantly differ between the three groups until age 18, nor did sedentary behavior show a significant influence (compare Figure 4). We therefore suppose the additional posture training to be the main contributor to the group-specific posture improvement.

Studies that examined the effect of muscular training programs on posture in adolescents are sparse. Interrelationships between muscle strength and posture parameters were found by Barczyk-Pawelec et al. (2015), but they did not examine the direct influence of muscle strength on posture. Kim et al. (2006) found that an imbalance in trunk muscle strength could influence lumbar lordosis, which they assume to be a risk factor for low back pain. Pure maximum strength building of the core-stabilizing musculature does not necessarily lead to an improved posture. Studies by Klee showed an effectiveness of a combined strength and stretch program on the body posture of adolescents, but only few parameters and their changes were analyzed (Klee, 1994). Similar results were presented by Park et al. (2014) who could improve multiple posture deviations by a 6 months training program. Their program consisted of strengthening and stretching exercises, but they did not include body perception exercises. Other studies only focused on thoracic kyphosis and outlined its improvement by an athletic training (Betsch et al., 2015). Bansal et al. (2014) summarized in a review that exercises may result in a modest improvement of (thoracic) posture. However, their results were not homogeneous, as some studies could not identify any effect (Bansal et al., 2014). The multidimensional training program completed for this study focused on stretching, targeted strength training, and body perception. Special emphasis was placed on the area of senso-neuromuscular coordination, in which the perception of the own body position (Maravita et al., 2003) and, in particular, the conscious change of the pelvic position, was exercised. It is assumed that the joint positions subconsciously readjust via a neuromuscular balance, which leads to a change of habitual posture. Apart from that, a conscious posture correction is enabled when muscles are purposefully controlled (active posture). This, however, requires a good proprioceptive perception of joint positions, such as the pelvic position, and targeted and controlled muscle activation.

The participants of the control group exhibited a mean athletic activity of between 1.7 and 1.9 h/week during adolescence. This is comparable to the “basic” athletic activity (i.e., hours of independent weekly training besides the posture training) of both training groups. Nevertheless, we are not able to answer the question of whether the supplementary training (2 h per week) of the training groups *per se* was responsible for posture improvement, or whether the specific exercises were the reason. In other words, we cannot safely conclude that an unspecific training program of two supplementary hours per week would not have produced a comparable effect. Athletic activities of only 2 h per week, as found in the control group until the age of 17 (Figure 4B) obviously were not able to improve posture significantly. Other studies confirmed the benefit of a special posture training program, as well (Klee, 1994; Park et al., 2016). Our results are also in accordance with D’Amico and colleagues who found that self-correction maneuvers producing an improvement of body posture have to be learned with specific postural training (D’Amico et al., 2017). According to this, we assume that our multidimensional concept was the main reason why we were able to find such clear improvements. In an earlier study we confirmed the short-time effect of sensorimotor exercises as performed in the present study on posture improvement (Ludwig et al., 2016a). Other studies only examined short-term training programs. Our study was designed to find possible long-term effects, and we already identified significant effects after just 2 years. In general, positive effects of strength training will occur after three to 6 months (Klee, 1994; Ludwig et al., 2016a). In the present study, significant effects occurred later, but we must be aware that the 1st months of strength training were in part performed with very low weights. We initially trained 14-year-old children who had to learn the correct movements first. Simultaneously, they had to develop an awareness to be able to rate the strain on a modified Borg scale. This process developed slowly, and was even slowed down by the trainer sometimes in order to prevent any kind of physical overload. After 1 year, we were sure that all participants were able to perform their training in a targeted manner. Therefore, it is comprehensible why significant changes occurred only after 1 or 2 years.

### Long-Term Effects in Adulthood

**FIGURE 5:**
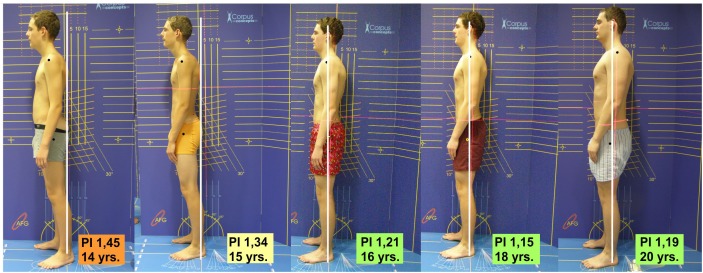
Example of posture development in a continuously exercising test person over a period of 6 years. Posture index (PI) values < 1.30 indicate a stable body posture. Courtesy of Elsevier publishing house.

Our second hypothesis, i.e., that posture training regularly practiced in adolescence carries positive effects on body posture into adulthood, could be confirmed only in parts. In the continually exercising group (TR20), the habitual posture constantly remained in a good range (HI = 1.27 ± 0.06). Figure 5 shows an example. In the group that stopped exercising from age 18 (TR18), the habitual posture index increased again. At the age of 20, it even deteriorated back to the initial value measured at the start of the study.

We interpret these findings that, if practiced continually, targeted posture training can sustainably improve the subconscious body posture. Since the habitual posture is maintained by means of subconsciously controlled neuro-motor processes (Sousa et al., 2012) and therefore decides on the strain placed on the musculoskeletal system in many everyday situations, it is especially important in terms of health aspects. More recent studies that identified an interrelation between posture deviations and the occurrence of back complaints in children and adolescents support this proposition (Dolphens et al., 2012, 2015, 2016). At a value of up to 65%, the prevalence of back pain in adolescence is noteworthy. To maintain a stable habitual posture, permanent training seems to be required.

In the continually exercising group (TR20), the active posture remained in a constant, good condition from age 16. The group that terminated their training from age 18 (TR18) also exhibited a good active posture, which differed significantly from the control group, but not from the TR20 group. We therefore conclude that the ability to control posture in a target-oriented manner seems to be preserved even after training breaks. Since in this posture position, a targeted deliberate muscular activation occurs, the development of a posture regulation skill can be viewed as the result of a learning process, during which not only strength endurance and flexibility were improved, but also the perception of one’s own body and the targeted muscular activation (sensorimotor control) (Maurer et al., 2006; D’Amico et al., 2017). Once learned, the motor programs required for this (i.e., targeted muscle activation in terms of time and amplitude) can be preserved over many years. Similar examples are known in swimming, biking, and skiing (Furley and Memmert, 2010). Like so many learned skills, their movement programs are presumably saved in the so-called ‘procedural memory’ (Squire, 2004). Once learned, these motor skills can be called upon after years without regular training being required. Therefore, we conclude that posture training performed in adolescence sustainably improves the ability of the test persons to correct their posture in a target-oriented and conscious manner in adulthood. We interpret this as positive (learning) effects that are carried over into young adulthood. However, the effects described are reproducible only when including the visual sense.

Comparing the active posture with closed eyes with the active posture with open eyes supplies an additional statement on the sensory information processing of the visual sense. When standing with closed eyes, body posture is controlled exclusively by proprioceptive sensory perception (Mallau et al., 2010; Chiba et al., 2016). Since the quality of posture regulation highly depends on targeted muscle control based on proprioception of the body, it needs to be trained.

The continually exercising group TR20 was able to maintain stable good posture values even without visual sensory information (i.e., their eyes were closed). Meanwhile, after the training stop of the TR18 group, a significant deterioration of their active posture occurred as soon as they closed their eyes. If posture deteriorates without the visual sense, the CNS is obviously not able to compensate for this loss in terms of posture control, e.g., by using only proprioceptive signals. Therefore, the changes of posture when closing the eyes give us a clue about the extent to which the CNS relies on the visual sense. It is known that proprioceptive information processing can be improved with exercises that are based on a targeted movement of certain body parts (e.g., the pelvic position) without vision (Bruhn et al., 2004; Zech et al., 2010). Such exercises were part of the weekly training program (see Table 2). Therefore, we interpret the fact of posture deterioration with closed eyes as suboptimal information processing of proprioceptive signals (Maurer et al., 2006; Mallau et al., 2010). That is, the CNS is not able to compensate the “switched off” visual sense by means of other sensory signals. According to our results, one may assume that this part of neuromuscular control obviously requires constant consolidation and is subject to deterioration if no adequate athletic activity takes place. This would explain why posture with closed eyes deteriorates in TR18 from the time when posture training was stopped. This finding corresponds to fundamental studies that had analyzed the adaptability of the neuromuscular control system (Asslander and Peterka, 2014). The CNS obviously changes the weighting of various sensor systems depending on the requirements in daily routine (Chiba et al., 2016). This could explain why a deterioration of posture regulation was observed without the visual sense when sensorimotor training was suspended. Nevertheless, we must be careful in interpreting these results as it is known that leisure behavior with strong visual components (e.g., playing computer games) may also influence visual signal processing in the brain (Oei and Patterson, 2013) and might also have confounded our results.

For practice, we can conclude that the proprioceptive perception of the body’s position without the use of the visual sense should be trained regularly.

Interrelationships between poor posture and back problems are known. From a medical-preventive point of view, targeted posture training should therefore start in early adolescence and be continued throughout life. Adequate training elements can be found in many types of sport, particularly in martial arts, gymnastics, and gymnastic disciplines in general (Perrin et al., 2002; Tsaklis et al., 2008; Zech et al., 2010; Iunes et al., 2016). In principle, every athletic training program can be supplemented by the corresponding elements. The training program described in this study is set in a ‘fitness sport’ environment and enjoys the advantage of a high degree of acceptance, especially in the group of adolescents. Adolescence is the time when classical sports clubs usually lose their appeal and gym training becomes more attractive (Aarnio et al., 2002; Tammelin et al., 2003). Qualified exercise instructions are, of course, an important precondition.

## Limitations

According to our knowledge, this study is the first that analyzed posture development and its trainability over a period of many years from adolescence to adulthood. However, it does have its limitations. For example, only symptom-free male test persons were examined. Statements on intervention options for back pain patients can therefore not be given. Nevertheless, from a preventive point of view, the target group with an increased pelvic tilt seems to be important, as students with posture weakness seem to have a greater prevalence of back pain in later adulthood (Dolphens et al., 2015).

### Group Allocation and Dropouts

As mentioned above, group allocation was not random. Though, we asked for the reasons why potential participants did not want to join the training group. Most of them did not refuse strength training on principle, but stated time and logistics reasons. We therefore assume that the control group did not consist of some sort of a “negative selection,” at least at the beginning of the study. At that time, their athletic activity and their sedentary time was comparable to both training groups (see Figure 4). However, at the end of the study, at age 20, they spent more time in a sedentary position than the training groups. Athletic activity of the control group was also going down at the end of the study. The fact that it did not reach significance level was possibly due to the low sample size and the high standard deviation.

Dropouts during such a long study period can hardly be avoided. We are aware that dropouts may produce a bias in the sense that unmotivated test persons leave the study at an early stage, leaving motivated test persons in the study and therefore generating more positive results. Although we could not totally avoid such a bias, we tried to keep its influence low. We included all test persons in our linear mixed model until the time of their dropping out. Furthermore, we asked in detail for the reasons of leaving the study. Most of them primarily stated logistics reasons or changes in their life situations (e.g., changing the place of residence, logistics problems/residence too far away from the fitness center, or too little time caused by their school situation or an apprenticeship). Only four participants left the study at age 18 because they had reached their individual training goals. All other participants of the TR18 group stated logistics reasons, as explained above. Therefore, we would not suggest a lack of motivation in any of these cases and view the resulting *bias* as acceptable.

### Posture Measurement

In general, habitual posture shows a daily fluctuation depending, for example, on a subject’s awareness and/or exhaustion. Earlier studies showed that the reproducibility of posture index measurements was good (Cronbach’s alpha = 0.842, Ludwig et al., 2016b). In addition, we tried to improve the internal validity of posture measurements by means of a standardized measuring protocol. Furthermore, all measurements were performed in the morning when the participants were not physically or mentally fatigued. Nevertheless, we cannot fully exclude posture fluctuations as a potential source of bias.

### Confounders

Even though potential disturbance variables, such as individual standing and sedentary behavior and supplementary athletic activities were surveyed using a questionnaire, they cannot entirely reflect differences in individual lifestyles. We did not find sedentary behavior to be a significant influencing factor during the course of the study. Nevertheless, we are aware that leisure behavior, that cannot fully be captured, might have had an influence on our output variables. Despite the control of potential disturbance variables, more complex factors that influence body posture, such as sporadic athletic activity or job-related factors, coming into play in adulthood, cannot be captured completely. We tried to evaluate possible influencing factors using a linear mixed model. Nevertheless, we are aware that there may be further factors that we are not aware of.

Since all test persons were supervised by one and the same tester, information regarding changes in leisure activity or additional athletic activity could be obtained between the yearly questionnaire surveys. For example, there was no test person who performed any additional athletic activities with strong balance components or sensorimotor training stimulus (e.g., gymnastics, skating, or martial arts), which could notably have improved their posture control. The “basic” athletic activity of all three groups was comparable, with most of the participants playing soccer, handball or performing cycling. In our yearly questionnaire we asked for the percentage of sensorimotor training or athletic training that in some sport clubs complements the practical training. Nevertheless, this factor was not susceptible of qualitative analysis, because we did not know the content and quality of these training sessions.

## Conclusion

Supplemental targeted athletic training of 2 h/week during adolescence may improve the active straightening of the body posture in a pain free population. This ability transitions into adulthood, even if training is no longer regular. Subconscious body posture (habitual posture) and posture with closed eyes, both regulated particularly by proprioceptive receptor systems, can also be improved by supplemental adequate training, but do require continuous exercising if the improvement is to be maintained.

## Ethics Statement

This study was carried out in accordance with the recommendations of the Saarland University Germany with written informed consent from all subjects and their parents, in accordance with the Declaration of Helsinki. The protocol was approved by the Ethic committee of Faculty 5 - Empirical Human Sciences.

## Author Contributions

OL, AH, and ES participated in the design of the study, carried out the experiments, and performed data analyses. OL wrote the manuscript. MF and AH helped to write the manuscript and supervised statistical analysis. JK contributed to the study design and supervised the entire project. All authors read and approved the final manuscript.

## Conflict of Interest Statement

The authors declare that the research was conducted in the absence of any commercial or financial relationships that could be construed as a potential conflict of interest. Parts of the data were published in German in a preliminary study in Sports Orthopedics/Sports Traumatology (doi: 10.1016/j.orthtr.2016.10.008). The approval for using individual data and graphics was provided by Elsevier publishing house.
