# Supplementary material for: Targeted Athletic Training Improves the Neuromuscular Performance in Terms of Body Posture From Adolescence to Adulthood – Long-Term Study Over 6 Years
Source: Front Physiol. 2018 Nov 27;9:1620. doi: 10.3389/fphys.2018.01620 (PMC6277893; doi:10.3389/fphys.2018.01620)
Supplement: Supplementary file 1 [file Data_Sheet_1.PDF]

QUESTIONNAIRE (Translation from German)

Name: ______ Prename: ______ Code: ______ Date: ______.

Weight: ______ kg Height: ______ cm

For minors: please fill out the questionnaire together with your parents.

(1)

Within the last 12 months …

… how many hours per week did you spent in a sedentary position (e.g., during school, study, homework, watching TV, playing video games)? Please indicate an average value.

about ______ hours (please round to half an hour, e.g. "2,5 hours")

(2)

Did your sedentary time change during the last 6 months (e.g. because you changed school or class)?

☐ No

☐ Yes

if you checked YES:

Within the last six months …

… how many hours per week did you spent in a sedentary position (e.g., during school, study, homework, watching TV, playing video games)?

about ______ hours (please round to half an hour)

(3)

Within the last 12 months …

… how many hours per week did you spent in a standing position (e.g., during school, homework, apprenticeship, work)? Please indicate an average value.

about ______ hours (please round to half an hour)

Did your standing time change during the last 6 months (e.g. because you changed school, began an apprenticeship, work, etc.)?

☐ No

☐ Yes

if you checked YES:

Within the last six months …

… how many hours per week did you spent in a standing position (e.g., during school, homework, apprenticeship, work)?

about ______ hours (please round to half an hour)

(4)

Apart from the athletic training in the fitness center, which type of athletic activity did you perform within the past year, how many hours per week did you perform this activity, and when did you first start with this athletic activity?

Athletic activity	Hours per week (including matches)<br>Please round to half an hour.	Started in
Examples:		
Soccer	*5*	*June 2001*
Inline-Scating	*3,5*	*Jan. 1998*
Your activities:		

☐ I did not perform any additional athletic activity.

(5)

If you performed ball sports, did you perform a specific athletic training within the framework of your ball sports training?

☐ No

☐ Yes

if you ticked YES, how many hours per week (rounded to half an hour) did you participate in a specific athletic training?

about ______ hours (please round to half an hour)
